# A cost‐effectiveness analysis of breast cancer treatment in certified versus non‐certified hospitals in Germany

**DOI:** 10.1002/ijc.70388

**Published:** 2026-03-09

**Authors:** Min‐Wai Lwin, Olaf Schoffer, Christoph Streissnig, Pauline Wimberger, Michael Gerken, Veronika Bierbaum, Christoph Bobeth, Martin Rößler, Patrik Dröge, Thomas Ruhnke, Christian Günster, Kees Kleihues‐van Tol, Theresa Link, Anton Scharl, Elisabeth C. Sturm‐Inwald, Karin Kast, Thomas Papathemelis, Olaf Ortmann, Monika Klinkhammer‐Schalke, Jochen Schmitt, Michael Schlander

**Affiliations:** ^1^ Division of Health Economics German Cancer Research Center (DKFZ) Heidelberg Germany; ^2^ Medical Faculty Mannheim University of Heidelberg Mannheim Germany; ^3^ Center for Evidence‐Based Healthcare, Faculty of Medicine and University Hospital Carl Gustav Carus TUD Dresden University of Technology Dresden Germany; ^4^ Klinik und Poliklinik für Frauenheilkunde und Geburtshilfe Universitätsklinikum Carl Gustav Carus an der Technischen Universität Dresden Dresden Germany; ^5^ National Center for Tumor Diseases (NCT), NCT/UCC Dresden, a partnership between DKFZ, Faculty of Medicine and University Hospital Carl Gustav Carus TUD Dresden University of Technology, and Helmholtz‐Zentrum Dresden‐Rossendorf (HZDR) Dresden Germany; ^6^ Tumorzentrum Regensburg Zentrum für Qualitätssicherung und Versorgungsforschung der Universität Regensburg Regensburg Germany; ^7^ Quality and healthcare research team Wissenschaftliches Institut der AOK (WIdO) Berlin Germany; ^8^ Arbeitsgemeinschaft Deutscher Tumorzentren e. V Berlin Germany; ^9^ Onkologische Fachklinik Bad Trissl Oberaudorf Germany; ^10^ Department of Gynecology and Obstetrics University Medical Center Regensburg Regensburg Germany; ^11^ Comprehensive Cancer Center Alliance WERA (CCC WERA) Regensburg Germany; ^12^ National Center for Tumor Diseases (NCT) Dresden Germany; ^13^ Zentrum Familiärer Brust‐ und Eierstockkrebs und Centrum für Integrierte Onkologie Universität zu Köln Köln Germany; ^14^ Klinik für Frauenheilkunde und Geburtshilfe Klinikum St. Marien Amberg Amberg Germany

**Keywords:** breast cancer, breast cancer treatment cost, cancer center certification, certified hospitals, cost‐effectiveness analysis

## Abstract

In Germany, the German Cancer Society (Deutsche Krebsgesellschaft [DKG]) accredits hospitals to ensure high‐quality cancer treatment through adherence to clinical guidelines and a multidisciplinary approach. Evidence suggests certified hospitals (CHs) achieve better clinical outcomes and prognoses than non‐certified hospitals (NCHs). However, additional services required for certification incur substantial, unreimbursed costs, necessitating a focused cost‐effectiveness evaluation. This retrospective cohort study utilized anonymized administrative routine healthcare data from Allgemeine Ortskrankenkasse, Germany's largest statutory health insurance. The study sample comprised 143,720 incident breast cancer (BC) patients treated between 2009 and 2017 across both CHs and NCHs. A health system perspective was used in this cost‐effectiveness analysis. Direct medical costs (inpatient, outpatient, medication, and certification) were compared between CHs and NCHs. Life‐years gained (LYG) were calculated from 5‐year restricted mean survival time. The incremental cost‐effectiveness ratio (ICER), quantified as cost per LYG, served as the primary outcome measure, reported in 2024 euro. Treatment in CHs significantly improved breast cancer survival, yielding 201 LYG per 1000 patients (95% confidence interval: 185–216). Accounting for €1.5 M in certification‐related costs and marginal direct medical costs, the total incremental cost was €1.81 M per 1000 patients. This resulted in an ICER of €9036 per LYG. Despite the financial investment required for DKG certification, BC treatment in CHs provided significant survival benefits at a reasonable incremental cost, reinforcing the clinical and economic value. These findings offer critical insights for hospital authorities and healthcare policymakers, supporting the continued investment in certification.

## What's New?

This study is the first to quantify the cost‐effectiveness of breast cancer treatment in German Cancer Society‐certified hospitals using real‐world data from over 140,000 patients. Analysis on certification‐related costs and survival outcomes demonstrates that high‐quality multidisciplinary care at certified hospitals delivers substantial clinical benefit at a reasonable incremental cost. The findings provide robust economic justification for hospital certification programs and offer valuable evidence to inform healthcare policy and reimbursement decisions.

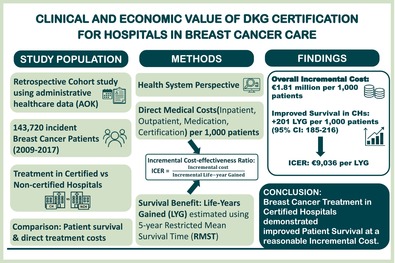

**Abbreviations**
€: euro
AOK: Allgemeine Ortskrankenkasse (Germany's largest statutory health insurance company)
ATC: Anatomical Therapeutic Chemical classification system
BfArM: Federal Institute for Drugs and Medical Devices
C: Organ‐Specific Cancer Centers
CC: Oncological Centers
CCC: Comprehensive Cancer Centers
CEACs: cost‐effectiveness acceptability curves
CHs: certified hospitals
CI: 95% confidence interval
DKG: German Cancer Society (Deutsche Krebsgesellschaft)
DKH: German Cancer Aid (Deutsche Krebshilfe)
DSA: Deterministic Sensitivity Analysis
EBM: The Uniform Evaluation Standard (Einheitlicher Bewertungsmaßstab)
ER: estrogen receptor
GDPR: General Data Protection Regulation
G‐DRG: German Diagnosis‐Related Group
HER2: human epidermal growth factor receptor 2
ICD‐10: 10th Revision of the International Classification of Diseases
ICER: incremental cost‐effectiveness ratio
InEK: The Institute for the Hospital Remuneration System
KBV: The National Association of Statutory Health Insurance Physicians (Kassenärztliche Bundesvereinigung)
LYG: life‐years gained
NCHs: non‐certified hospitals
OPS: Operations and Procedure Classification System
OS: overall survival
PR: progesterone receptor
PZN: Pharmaceutical Central Number
RMST: restricted mean survival time
RWD: real‐world data
SA: sensitivity analysis
SE: standard error
SHI: statutory health insurance
WiZen: Effectiveness of Care in Certified Cancer Centers Study (Wirksamkeit der Versorgung in onkologischen Zentren)
WTP: willingness‐to‐pay
ZfKD: German Centre for Cancer Registry Data (Zentrum für Krebsregisterdaten)

## 1 INTRODUCTION

Breast cancer (BC) is the most common cancer and the leading cause of cancer‐related death among women worldwide.^1^ In 2022, approximately 74,500 women were diagnosed with BC (10th Revision of the International Classification of Diseases [ICD‐10] C50) in Germany, and around 6000 were diagnosed with carcinoma in situ (ICD‐10 D05). This incidence rate equates to one in eight women in Germany being diagnosed with BC in their lifetime. With approximately 315,000 patients living with BC (5‐year prevalence), the disease claims over 18,500 lives each year in Germany, according to the German Centre for Cancer Registry Data (Zentrum für Krebsregisterdaten [ZfKD]).^2^

Given the large patient population, BC has significant economic and healthcare policy implications. In 2020, BC in Germany cost approximately €4.42 billion in medical expenses.^3^ The highest contributors to this economic burden include inpatient care, medication, and productivity losses.^4^ In recent decades, advances in BC screening and therapy—particularly the introduction of personalized, targeted multimodal treatments—have significantly improved long‐term survival.^5^ BC treatment options include surgery, radiotherapy, endocrine therapy, chemotherapy, immunotherapy, and further targeted therapies. Treatment and prognosis are determined by factors such as age, cancer stage, and tumor characteristics, including estrogen receptor (ER), progesterone receptor (PR), human epidermal growth factor receptor 2 (HER2) status, histologic grade, and the proliferation marker Ki67.^6^ With the growing complexity of BC treatments due to advancements in diagnostics, surgery, and medical technology, it is crucial to adhere to standardized treatment protocols and guidelines, ensuring consistent and evidence‐based care for better patient outcomes and reduced variability in treatment approaches.

The German Cancer Society (Deutsche Krebsgesellschaft [DKG]) provides the certification and auditing of hospitals in Germany. BC was the first tumor entity included in the certification program since 2003.^7^ In accordance with Goal 5 of the National Cancer Plan, the National Certification Program has established a three‐tier oncological care model. This includes Organ‐Specific Cancer Centers (C), which focus on specific tumor types, and Oncological Centers (CC), which provide care for multiple cancer types. Both C and CC centers are certified by the DKG. Comprehensive Cancer Centers (CCC) are co‐certified by DKG and German Cancer Aid (Deutsche Krebshilfe [DKH]) and mostly affiliated with university hospitals, combining multidisciplinary patient care with cancer research and the development of new treatment approaches.^7^

Hospitals can apply for certification on a voluntary basis. This is often because they have the necessary resources and infrastructure to meet the certification criteria, as well as the capacity to cover the associated costs and effort. Consequently, some hospitals do not seek certification. These hospitals also treat BC patients, as patients in Germany have a legal right to choose where to receive treatment. Patients' decisions about where to receive treatment can therefore be influenced by a variety of subjective and objective factors, in addition to certification.

Certified hospitals (CHs) must adhere to defined quality standards in areas such as structural organization, diagnostic procedures, and treatment methods, including breast surgery, pharmacological therapies, radiotherapy, and psychosocial support. Certification and recertification, conducted every 3 years, require compliance with national treatment guidelines, the establishment of structured patient pathways, and the organization of interdisciplinary tumor boards to facilitate interdisciplinary decision‐making, etc. As of 2024, 257 hospitals are DKG‐certified for BC treatment (Source: German Cancer Society, DKG).^8^

Patient prognosis is a key indicator of cancer quality care and clinical effectiveness. Several studies suggest that care provided by CHs is associated with improved short‐ and long‐term survival.^9^, ^10^, ^11^ A multi‐institutional research initiative, “Effectiveness of Care in Certified Cancer Centers study (Wirksamkeit der Versorgung in onkologischen Zentren, WiZen),” further supports this observation, demonstrating superior clinical outcomes and significantly improved patient survival rates in CHs compared to non‐certified hospitals (NCHs).^12^, ^13^, ^14^

Despite the additional costs associated with meeting certification requirements, the costs incurred by hospitals were not eligible for reimbursement within the German Diagnostic‐Related Group (G‐DRG) framework. A 2017 report by Prognos AG estimated these annual costs to range from €0.2 to €10.4 million, depending on hospital size and DKG certification type.^15^ These unreimbursed costs raise questions about overall cost‐effectiveness and necessitate further investigation into the cost‐effectiveness of BC treatment in CHs. A research by Chen et al.^16^ indicates that CHs can provide more cost‐effective care in colon cancer treatments, suggesting the potential for similar benefits in BC.

Our research will examine the cost‐effectiveness of BC care in DKG‐certified versus NCHs, using an administrative dataset from a German statutory health insurance (SHI). By analyzing resource utilization and survival outcomes, we aim to generate updated evidence and conduct a cost‐effectiveness analysis from a health system perspective, considering both direct treatment costs and center certification costs. The findings will provide health economic insights to inform policymakers and guide future decisions regarding DKG certification to hospitals.

## 2 METHOD

### 2.1 Data source

Our retrospective cohort study utilized anonymized billing data from Germany's largest statutory health insurer, Allgemeine Ortskrankenkasse (AOK), covering 2009–2017. This administrative data provided essential information for cost analysis and patient survival. Our cost‐effectiveness analysis was based on a cohort of BC patients from the WiZen project, which investigated survival outcomes across 11 cancer types by comparing DKG‐certified and NCHs in Germany.

The study sample included AOK‐insured individuals aged 18 or older diagnosed with incident BC (ICD‐10: C50 and D05) between 2009 and 2017. An index treatment was defined as the first inpatient treatment for a primary or secondary BC diagnosis. Exclusion criteria included coinciding diagnosis and death dates, absence of an inpatient primary diagnosis, changes in center status, zero survival time, missing hospital characteristics, non‐continuous AOK insurance, primary resection more than 6 months post‐index treatment, or treatment at hospitals within 1 year before DKG certification.

The final cohort of 143,720 BC patients was divided into two groups: patients initially treated in CHs versus NCHs. This grouping enabled comparative analysis of treatment outcomes and economic implications between certified and non‐certified facilities. Details of the data preparation process can be found in Supporting Information S1: Appendix A.

### 2.2 Survival analysis for evaluation of the life‐year gained

Firstly, we performed survival analysis using the Kaplan–Meier method to assess overall survival (OS) in BC patients treated at CHs and NCHs. A 5‐year survival analysis was used for comparison with other studies. Survival time was measured from the index treatment date to death or censoring. The restricted mean survival time (RMST), representing average survival within 5 years, was derived by integrating the area under the Kaplan–Meier curve. RMST difference provided an intuitive measure of survival benefit, handling censored data without relying on proportional hazards assumptions. OS reflected the combined impact of all treatment qualities (surgery, chemotherapy, radiotherapy, endocrine therapy, and supportive care), making it useful for evaluating overall effectiveness.

To adjust for potential confounding factors, we fitted a Cox proportional hazards regression model. This model enabled adjustments for age group (18–59, 60–79, and 80+), metastatic status (ICD‐10 C78, C79), comorbidity (Elixhauser Comorbidity Index), and hospital size (1–299, 300–499, 500–999, and 1000+ beds). RMST of Cox model was then calculated by numerically integrating these adjusted survival curves, providing an adjusted estimate of confounder impact. Bootstrapping (10,000 samples) yielded mean and 95% confidence interval estimates for the Cox model's RMST.

Our study utilized Microsoft Excel and R version 4.1.2 for data processing, statistical analysis, and graphical representation.

### 2.3 Calculation of reimbursement costs

The cost of a disease on a patient population could be estimated using two primary methods: micro‐costing or top‐down methods.^17^ The top‐down approach allocated macro‐level healthcare expenditures based on assumptions. In contrast, the micro‐costing method provided greater precision by calculating patient‐level costs within a selected sample to determine average and total expenditures. Evaluation of BC cost required identifying costs directly associated with BC treatment while considering the impact of comorbidities. In our study, micro‐costing from the administrative/claim data was used to estimate the direct cost incurred by BC patients.^18^

**FIGURE 1:**
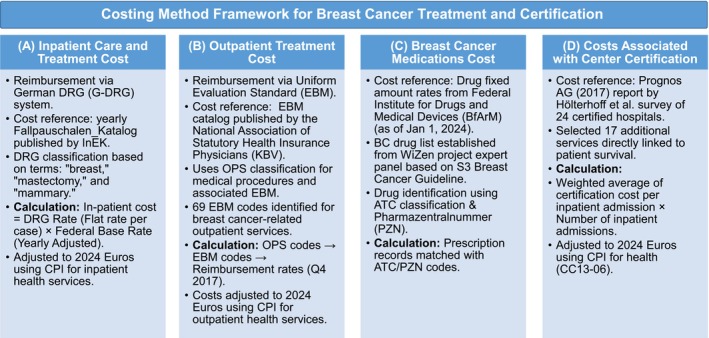
Overview of cost calculation framework for breast cancer treatment and certification. (A) Inpatient care and treatment cost. (B) Outpatient treatment cost. (C) breast cancer medications cost. (D) Costs associated with center certification. ATC, Anatomical Therapeutic Chemical Classification System); BfArM, Federal Institute for Drugs and Medical Devices; CPI, Consumer Price Index; EBM, Einheitlicher Bewertungsmaßstab (Uniform Evaluation Standard); G‐DRG, German Diagnosis‐Related Groups system; InEK, Institute for the Hospital Remuneration System; OPS, Operationen‐ und Prozedurenschlüssel (Operations and Procedure Classification System); PZN, Pharmazentralnummer (Pharmaceutical Central Number.

While total patient counts represented incident BC cases, individuals might have multiple records within the inpatient and outpatient treatment records due to repeated visits over their 5‐year survival period following index treatment. This incident costing method followed a patient‐level, longitudinal approach, tracking the actual resource utilization and healthcare expenditures incurred throughout the disease trajectory. It reflected the total financial burden accrued throughout the study period, particularly relevant for survival analyses. The incident costing did not support annualized analysis but aligned with the use of survival curves, RMSTs, and other longitudinal measures. A detailed costing method framework for included cost categories in our study was described in Figure 1.

#### 2.3.1 Inpatient care and treatment cost

In Germany, health care providers (hospitals/treatment centers) were reimbursed with a lumpsum amount for each inpatient admission through a fixed amount payment system.^19^ This system relied on the G‐DRG classification, which standardizes reimbursement based on the diagnosis and treatment provided to patients. The Institute for the Hospital Remuneration System (InEK) was responsible for annually publishing a fixed amount catalog (Fallpauschalen_Katalog) that serves as a cost reference for these payments. In this study, DRGs related to BC and corresponding inpatient admissions were identified using search terms such as “breast,” “mastectomy,” and “mammary,” resulting in 38 BC‐related DRGs. A breakdown of total admissions for each BC‐related DRG, including descriptions, can be found in Supporting Information S1: Appendix B.

The DRG rate (flat rate per case) of each admission was multiplied by the federal base rate of each year to obtain the average reimbursement in euro. We acknowledge that DRG rates were subject to additional cost adjustments based on admission duration and hospital performance. Thus, the national average reimbursement rates for each DRG were used to estimate the average BC treatment costs (Equation 1). The cost data were truncated at 5 years from the index treatment for each patient to calculate reimbursement for each BC patient during their 5‐year survival period. The reimbursements were adjusted to 2024 euro using the consumer price index of inpatient health services for Germany.

(1) $$\text{Reimbursement amount}=\text{flat rate}\;\mathrm{per}\;\text{case}\;\left(G-\mathrm{DRG}\right)\times \text{federal base rate}\;\left(\text{Bundesbasisfallwert}\right).$$

Example:

Inpatient admission with G‐DRG J23Z.

(Description: major operations on the breast in the case of a malignant neoplasm).

Average flat rate per case for G‐DRG J23Z in 2017 = 1.495.

Federal base rate of the year 2017 = €3376.11.

Reimbursement amount for health care provider = 1.495 × 3376.11 = €5047.28 (2017).

#### 2.3.2 Outpatient treatment cost

The Uniform Evaluation Standard (Einheitlicher Bewertungsmaßstab‐EBM) catalog was a standardized framework for billing outpatient medical services within Germany's SHI system, ensuring that only accurately coded and documented services qualify for reimbursement.^20^ The National Association of Statutory Health Insurance Physicians (Kassenärztliche Bundesvereinigung‐KBV) published the EBM catalog as quarterly cost reference sheets.

The EBM catalog incorporated the Operations and Procedure Classification System (Operationen‐ und Prozedurenschlüssel—OPS), a classification system that provided standardized descriptions of medical procedures. The OPS table in the EBM catalog specified detailed codes for procedures and their corresponding services in outpatient settings, enabling precise billing for outpatient care.

For BC procedures, the OPS table listed the relevant OPS codes, corresponding EBM codes, and the allowable health services in outpatient care. A total of 69 EBM codes were identified as applicable to BC‐related outpatient services (see Supporting Information S1: Appendix C). The cost reference sheet from Q4 2017 was used to estimate reimbursements for BC patients in the cohort. These costs were adjusted to 2024 euro using the consumer price index of outpatient health services for Germany.

#### 2.3.3 Cost calculation for breast cancer medications

In Germany, pharmaceutical products were uniquely identified using the Anatomical Therapeutic Chemical (ATC) classification system and Pharmazentralnummer (PZN) codes. This system facilitated the conversion of prescriptions into standardized reimbursement amounts. Drawing upon clinical expertise and the S3 Breast Cancer Treatment Guideline, a panel of clinicians from the WiZen project established a list of 27 BC‐related drugs with their associated ATC codes.^6^ Medication costs were calculated from issue records in the dataset, using PZN and ATC codes and the fixed reimbursement rates published by the Federal Institute for Drugs and Medical Devices (BfArM) and market prices as of January 1, 2024.^21^ This methodology for estimating medication costs, anchored to a single latest official pharmaceutical reference prices, serves to mitigate distortions arising from market volatility and to neutralize potential pricing effects associated with the introduction of biosimilars. See Supporting Information S1: Appendix D for a detailed list of medications and their associated costs incurred by each patient group.

#### 2.3.4 Costs associated with center certification

Hölterhoff et al. identified the additional services and associated costs for German Cancer Society (DKG) certification through a survey of 24 CHs.^15^ This report categorized fixed and variable costs across different hospital levels. Of the 31 identified services, 6 were excluded as they pertain only to Comprehensive Cancer Centers (CCC), and 8 were deemed unrelated to patient survival. Consequently, 17 services, considered directly linked to patient survival based on Cheng et al.'s assumptions, were selected for further analysis (see Supporting Information S1: Appendix E).

To calculate the certification costs attributable to certified hospital patient group, we calculated a weighted average of the certification cost per inpatient, based on the cost estimates from Hölterhoff et al., and multiplied it by the number of inpatient admissions in our dataset. All costs were adjusted to 2024 euros using the German consumer price index for health (CPI; CC13‐06).

### 2.4 Cost‐effectiveness analysis

The incremental cost‐effectiveness ratio (ICER) measured the additional cost incurred per unit of health benefit (e.g., life‐years gained [LYG]) when comparing two healthcare interventions. It was calculated as the difference in costs divided by the difference in effect between the new and standard treatments/intervention. A lower ICER indicates better cost‐effectiveness, and it is often compared to a willingness‐to‐pay (WTP) threshold to determine if an intervention is economically viable. A 3% discount rate is applied on both cost and benefit.

The ICER between certified and NCHs was derived using the following equation^17^:

(2) $$\text{ICER}=\frac{\text{Incremental cost}\;}{\text{incremental life}-\text{year gained}}=\frac{\mathrm{Cos}t_{\mathrm{CH}}\;-\;\mathrm{Cos}t_{\mathrm{NCH}}}{\mathrm{RMS}T_{\mathrm{CH}}\;-\;\mathrm{RMS}T_{\mathrm{NCH}}},$$

where ICER = incremental cost‐effectiveness ratio, Cost_CH_ = average cost of BC treatment per 1000 BC patients in CHs, Cost_NCH_ = average cost of BC treatment per 1000 BC patients in NCHs, RMST_CH_ = restricted mean survival time per 1000 BC patients in CHs patient group, RMST_NCH_ = restricted mean survival time per 1000 BC patients in NCHs patient group.

### 2.5 Sensitivity analyses

We performed several sensitivity analyses (SA) to evaluate the robustness of our ICER results, specifically: discount rate variations (SA1: 0% and SA2: 5%), inclusion of all additional services in certification cost (SA3), a probabilistic sensitivity analysis (PSA) presented as a cost‐effectiveness plane, and a cost‐effectiveness acceptability curve (CEAC).

## 3 RESULTS

### 3.1 Patient characteristics

Table 1 summarizes patient demographics, tumor characteristics, survival outcomes, and hospital metrics, stratified by DKG certification status (certified: *n* = 91,269; non‐certified: *n* = 52,451). Patients treated at CHs were slightly younger at diagnosis (mean age 65.5 years, 95% CI: 63.6–67.4) compared to those at NCHs (mean age 67.1 years, 95% CI: 65.2–69.0), with CHs treating a greater proportion of patients aged 18–79 and NCHs treating more patients aged 80+. CHs also reported slightly higher rates of carcinoma in situ (8.7% vs. 7.1%) and distant metastasis (13.4% vs. 11.6%).

**TABLE 1:** Comparison of patient demographics, tumor characteristics, survival outcomes, and hospital metrics by hospital certification status.

Attributes	Hospital certification status	
	Yes (*n* = 91,269)	No (*n* = 52,451)
Patient demographics		
Mean age at index treatment	65.5	67.1
Age 18–59 years (%)	33.4	30.2
Age 60–79 years (%)	51.1	49.6
Age 80+ years (%)	15.6	20.2
Tumor characteristics		
Carcinoma in situ D05 (%)	8.7	7.1
Malignant neoplasm of breast C50 (%)	91.3	92.9
Distant metastasis C78/C79 (%)	13.4	11.6
Secondary oncological disease (%)	16.3	15.9
Mean Elixhauser Comorbidity Index	6.21	6.37
5‐Year survival and follow‐up statistics		
Mean follow‐up time (in year)	3.5	3.2
Deaths/events (*n*)	15,158	12,031
Deaths/events (%)	16.6	22.9
KM model (unadjusted)		
5‐Year overall survival probability (%)	78.3	71.9
RMST (in year, CI)	4.398 (4.389–4.408)	4.182 (4.168–4.195)
Life‐year gained (CI)^a^	0.216 (0.200–0.233)	Ref
RMST ratio (CI)^a^	1.052 (1.048–1.056)	Ref
Cox model (adjusted)		
5‐Year overall survival probability (%)	85.5	82.0
RMST (in year, CI)	4.632 (4.622–4.642)	4.539 (4.526–4.552)
Life‐year gained (CI)^a^	0.093 (0.082–0.104)	Ref
RMST ratio (CI)^a^	1.020 (1.018–1.023)	Ref
Hospital characteristics		
Total number of hospitals	280	730
1–299 beds (%)	21.8	64.5
300–499 beds (%)	32.1	24.2
500–999 beds (%)	30.7	10.1
1000+ beds (%)	15.4	1.1
Total inpatient admissions	146,014	83,900
Average inpatient per center	521	115
Average reimbursement per inpatient (€, CI)	5544 (5532–5556)	5287 (5270–5304)
Average length of hospital stay (days, CI)	6.08 (6.06–6.09)	6.15 (6.13–6.17)

Abbreviations: C78, secondary malignant neoplasm of respiratory and digestive organs; C79, secondary malignant neoplasm of other and unspecified sites; CI, 95% confidence intervals; KM, Kaplan–Meier; RMST, restricted mean survival time.

^a^ The NCHs group is the reference category.

A longer mean follow‐up (3.5 vs. 3.2 years), a higher 5‐year survival rate (78.3% vs. 71.9%), and a lower percentage of deaths/events (16.6% vs. 22.9%) were observed at CHs. Regarding hospital characteristics, CHs tended to be larger (500–999 beds: 30.7%, 1000+ beds: 15.4%), and slightly shorter hospital stays (6.08 vs. 6.15 days).

### 3.2 Life‐year gained in certified hospitals

**FIGURE 2:**
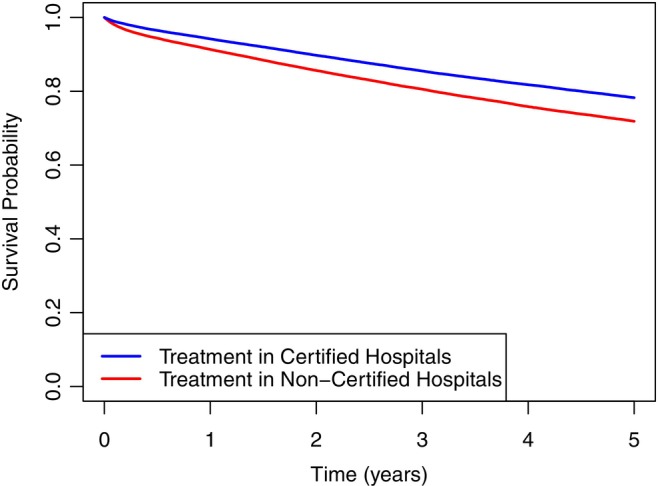
Kaplan–Meier survival curves of breast cancer patients in certified hospitals versus non‐certified hospitals.

Analysis of RMST revealed a statistically significant survival difference between patients treated at CHs and NCHs. With the Kaplan–Meier (KM) method, the mean RMST was 4.398 years (95% CI: 4.389–4.408) for CHs and 4.182 years (95% CI: 4.168–4.195) for NCHs, representing a gain of 0.216 life years on average (95% CI: 0.200–0.233) in CHs. The RMST ratio of 1.052 (95% CI: 1.048–1.056) indicated a 5.2% longer survival in CHs within the 5‐year timeframe. The survival graph for patients in CHs versus NCHs is presented in Figure 2.

After adjusting for age group, metastasis, comorbidity, and hospital size using a Cox model, the significant difference in RMST persisted. The adjusted RMST was 4.632 years (95% CI: 4.622–4.642) in CHs and 4.539 years (95% CI: 4.526–4.552) in NCH. This corresponded to a difference of 0.093 years (95% CI: 0.082–0.104) and a ratio of 1.02 (95% CI: 1.018–1.023), demonstrating a 2% longer survival in CH. These results suggest a modest but significant survival benefit associated with treatment at CHs, after accounting for potential confounders.

### 3.3 Breast cancer care and treatment cost

Patient population, inpatient admissions, outpatient treatments, and their associated costs from 2009 to 2017, stratified by hospital certification status is presented in Table 2. During this 9‐year period, CHs treated 91,269 patients (63.5%) and NCHs treated 52,451 patients (36.5%), for a total of 143,720 patients.

**TABLE 2:** Number of incident breast cancer cases, inpatient admissions, treatment costs, and certification costs by year and certification status.

Year	Number of patients			Inpatient Admissions			Inpatient treatment cost			Outpatient treatment cost			Certification cost
	CHs	NCHs	Total	CHs	NCHs	Total	CHs	NCHs	Total	CHs	NCHs	Total	CHs
2009	10,670	7916	18,586	18,572	13,798	32,370	€101,408,223	€71,428,088	€172,836,311	€1,365,131	€1,290,315	€2,655,446	€17,383,975
2010	10,615	6961	17,576	18,321	12,106	30,427	€101,540,579	€63,882,509	€165,423,087	€2,972,761	€2,075,793	€5,048,555	€17,149,032
2011	10,421	6564	16,985	18,219	11,074	29,293	€103,613,231	€60,968,090	€164,581,321	€4,702,600	€3,038,421	€7,741,021	€17,053,556
2012	10,091	6049	16,140	16,964	10,137	27,101	€91,734,392	€51,693,952	€143,428,344	€5,967,260	€3,499,612	€9,466,872	€15,878,837
2013	10,243	5707	15,950	16,742	9215	25,957	€91,420,003	€47,598,584	€139,018,587	€7,038,584	€3,979,455	€11,018,039	€15,671,038
2014	9918	5084	15,002	15,525	8012	23,537	€86,488,300	€42,331,224	€128,819,523	€7,612,315	€3,990,781	€11,603,095	€14,531,888
2015	9941	4948	14,889	15,058	7439	22,497	€85,330,643	€40,075,921	€125,406,564	€7,423,724	€3,567,292	€10,991,016	€14,094,761
2016	9610	4597	14,207	13,813	6393	20,206	€78,040,984	€35,061,598	€113,102,582	€7,239,352	€3,274,306	€10,513,658	€12,929,402
2017	9760	4625	14,385	12,800	5726	18,526	€69,927,805	€30,576,376	€100,504,181	€7,147,887	€3,273,214	€10,421,102	€11,981,202
Grand total	91,269	52,451	143,720	146,014	83,900	229,914	€809,504,158	€443,616,343	€1,253,120,501	€51,469,615	€27,989,189	€79,458,805	€136,673,691
Average per patient							€8869	€8458	€8719	€564	€534	€553	€1497

*Note*: Annualized analysis is not supported in this table. Patient counts reflect new (incident) breast cancer diagnoses, but individual patients may be counted more than once in inpatient and outpatient treatment records within 5 years of their initial (index) treatment. Certification costs were not reimbursable and applied only to patients in the CHs group. Reimbursed costs in each year were adjusted to 2024 euros using the German consumer price index for each health service type.

Abbreviations: CHs, certified hospitals; NCHs, non‐certified hospitals.

CHs managed a significantly larger proportion of inpatient admissions (63.5%, 146,014 admissions) compared to NCHs (36.5%, 83,900 admissions), totaling 229,914 admissions. This disparity was reflected in total inpatient treatment costs, with CHs accounting for €809.5 million (64.6%) and NCHs €443.6 million (35.4%), resulting in a cumulative cost of €1.25 billion. CHs exhibited a 4.9% increase in both average cost per patient (€8869 vs. €8458) and average cost per admission (€5544 vs. €5287) relative to NCHs. The higher patient and admission volumes, coupled with the slightly elevated costs, suggest potential variations in resource allocation and patient care strategies between the two hospital types.

In term of outpatient treatment costs, CHs accounted for a total of €51.47 million, while NCHs contributed €27.99 million, resulting in a combined total of €79.46 million. In line with the approximately 5% relative difference seen in other cost categories, the average outpatient cost per patient was 5.6% higher in CHs (€564) than in NCHs (€534).

The certification‐attributable costs over the study period amounted to €136.67 million for the CHs patient group, while no such costs were reported for NCHs. The certification‐attributable cost per patient was €1497, highlighting the financial resources incurred for CHs (see Table 2).

### 3.4 Breast cancer medication cost

Analysis of drug consumption and associated costs revealed several key findings reflecting BC medication within the cohort. Trastuzumab stands out as the most costly medication overall, with expenditures of €22,886,900 in CHs and €11,245,341 in NCH. While not as costly as targeted drugs, anti‐hormonal drugs show substantial utilization (number of issued medications) in both CHs and NCHs. Tamoxifen was the most frequently issued medication in this category. Chemotherapeutics show comparatively lower utilization and cost than targeted drugs and anti‐hormonal drugs.

Total medication costs for the cohort reached €134.4 million, with CHs accounting for €86.7 million (64.5%) and NCHs contributing €47.7 million (35.5%). While the overall average cost per patient was €935, per‐patient costs were slightly (4.4%) higher in CHs (€950) compared to NCHs (€910). Detailed drug consumption data are presented in Supporting Information S1: Appendix D.

### 3.5 Incremental cost per 1000 breast cancer patients

Summary of mean cost per 1000 patients (Table 3) compares the BC costs between CHs and NCHs across different categories, alongside an incremental cost analysis. Certification costs, which do not apply to NCHs, amount to €1.5 million in CHs. CHs demonstrated higher costs compared to NCHs in all areas: inpatient (€8.87 vs. €8.46 million), outpatient (€0.56 vs. €0.53 million), and medication (€0.95 vs. €0.91 million).

**TABLE 3:** Comparison of cost categories between certified hospitals (CHs) and non‐certified hospitals (NCHs) with incremental cost analysis.

Cost category	Mean cost in CHs (€) (95% CI)	Mean cost in NCHs (€) (95% CI)	Incremental cost (€) (95% CI)
Certification cost	€1,496,957 (1,495,177–1,498,737)	‐	€1,496,957 (1,495,177–1,498,737)
Inpatient cost	€8869,430 (8,849,468–8,889,395)	€8,457,730 (8,430,732–8,484,726)	€411,700 (378,124–445,276)
Outpatient cost	€563,930 (560,956–566,910)	€533,630 (530,485–536,765)	€30,300 (25,973–34,627)
Medication cost	€949,680 (942,180–957,185)	€909,820 (900,614–919,027)	€39,860 (27,983–51,736)
Total cost	€11,879,997 (11,858,390–11,901,604)	€9,901,180 (9,872,484–9,929,876)	€1,978,817 (1,942,896–2,014,737)

*Note*: Costs are presented in 2024 euros (€) (undiscounted) and represent the average cost per 1000 patients in each category. Certification costs apply only to certified hospitals and do not apply to non‐certified hospitals. Incremental cost was calculated as the difference between the mean costs of CHs and NCHs. The sum of variances method was used to compute the standard error (SE) and 95% confidence intervals for both the total cost across cost categories and the incremental cost between CHs and NCHs.

Abbreviation: CI, confidence interval.

The total healthcare cost per 1000 patients was €11.88 million in CHs and €9.90 million in NCHs. This resulted in an incremental cost of €1.98 million (95% CI: €1.94 to €2.01 million), indicating a notable cost difference between the two groups.

### 3.6 Cost‐effectiveness analysis: evaluating the incremental cost per life‐year gained

A comparative analysis for the total cost and life years of BC treatment in CHs versus NCHs is presented in Table 4. BC treatment in CHs had higher costs but better survival outcomes. The ICER was derived using Equation (2).

**TABLE 4:** Cost‐effectiveness of breast cancer (BC) treatment in certified versus non‐certified hospitals.

Analysis type	CH		NCH		Incremental cost	Incremental LYG	
	Total net treatment cost (€)	Total survival life years	Total net treatment cost (€)	Total survival life years			ICER (€/LYG)
Main analysis							
Kaplan–Meier model (17 additional services)							
Base: 3% discount rate	€10,881,382	4084	€9,068,901	3883	€1,812,481	201	€9036
SA1: 0% discount rate	€11,879,997	4398	€9,901,180	4182	€1,978,817	216	€9161
SA2: 5% discount rate	€10,286,834	3891	€8,573,386	3700	€1,713,448	191	€8965
Adjusted Cox model (17 additional services)							
Base: 3% discount rate	€10,881,382	4301	€9,068,901	4215	€1,812,481	86	€20,987
SA1: 0% discount rate	€11,879,997	4632	€9,901,180	4539	€1,978,817	93	€21,343
SA2: 5% discount rate	€10,286,834	4098	€8,573,386	4016	€1,713,448	82	€20,823
Sensitivity analysis: including all services							
Kaplan–Meier model (25 additional services)							
Base: 3% discount rate	€11,710,047	4084	9,068,901	3883	€2,641,146	201	€13,168
SA1: 0% discount rate	€12,784,711	4398	€9,901,180	4182	€2,883,530	216	€13,350
SA2: 5% discount rate	€11,070,222	3891	€8,573,386	3700	€2,496,836	191	€13,065
Adjusted Cox model (25 additional services)							
3% discount rate	€11,710,047	4301	€9,068,901	4215	€2,641,146	86	€30,583
SA1: 0% discount rate	€12,784,711	4632	€9,901,180	4539	€2,883,530	93	€31,100
SA2: 5% discount rate	€11,070,222	4098	€8,573,386	4016	€2,496,836	82	€30,343

*Note*: Results are based on a cohort of 1000 BC patients. Total net treatment costs are in euros. Sensitivity analyses used 0% (SA1) and 5% (SA2) discount rates on both cost and benefit. The adjusted model used the Cox proportional hazards model in survival analysis, adjusting for age group, metastasis, and hospital size to obtain new estimates of adjusted for each group.

Abbreviations: CHs, certified hospitals; ICER, incremental cost‐effectiveness ratio (€ per life‐year gained); LYG, life‐years gained; NCHs, non‐certified hospitals; SA, sensitivity analysis.

In the KM model analysis with a 3% discount rate, BC treatment in CHs cost €10.88 M versus €9.07 M in NCH, with 4084 versus 3883 life years, yielding an ICER of €9036 per LYG. SA (0% and 5% discount) showed ICERs of €9161 and €8965.

After adjusting for age group, metastasis, comorbidity, and hospital size using a Cox model, ICERs increased significantly (€20,987), indicating higher costs per survival year when adjustments were applied. At 0% and 5% discount rates, adjusted ICERs result in €21,343 and €20,823, respectively (see Table 4).

#### 3.6.1 Sensitivity analyses

The main analysis calculated certification costs by including only additional services directly impacting patient survival. A third sensitivity analysis (SA3) was included to evaluate the impact of including all 25 additional services in DKG certification. This led to a total cost increase in the CHs arm and a slight rise in the ICER, assessing the robustness of our results to broader cost considerations. In the KM analysis of SA3, the ICERs were €13,350 (0% discount rate), €13,168 (3%), and €13,065 (5%). In the adjusted Cox model, the ICERs were €31,100 (0%), €30,583 (3%), and €30,343 (5%), respectively (see Table 4).

For further SA of the cost‐effectiveness results, including probabilistic sensitivity analyses (PSA) and the probability of cost‐effectiveness at varying WTP thresholds, please refer to Supporting Information S1: Appendixes F–H.

## 4 DISCUSSION

To the best of our knowledge, this is the first study to evaluate the cost‐effectiveness of BC treatment in CHs in Germany. Our study assessed the direct treatment costs and LYG for BC treatment in CHs versus NCH. Despite a significant cost burden of obtaining and maintaining certification, we found that BC treatment in CHs, with only a modest increase in cost, provided substantial additional clinical benefits of 201 life years per 1000 BC patients over a 5 years survival period.

The rising healthcare expenditures, particularly the novel cancer therapies, are placing a significant strain on health systems. However, research on BC treatment costs in Germany remains limited. Gruber et al. (2012), using 1999 claims data adjusted to 2010 values, estimated average annual BC costs between €6000 and €10,000 per woman, with higher costs for younger patients.^22^ Kreis et al. reported age‐standardized, phase‐specific inpatient costs based on 2011–2014 claims data, including €4982 for initial care (11 months), €482 annually for intermediate care, and €15,488 for terminal care (11 months).^23^ Above studies considered all costs in the claim data including (remedies/aids, rehabilitation, sick leave, and travel, etc.), which our study did not. Similar total BC cost estimates were obtained by Khan et al. (2023) using a decision modeling approach.^24^ A systematic review by Franklin et al.^4^ identified a wide range of annual BC treatment costs in Europe (€151–€29,753). Direct comparison with other studies is difficult due to methodological variations and different cost categories considered. Healthcare system differences, health technology assessment processes, pricing, and national policies contribute to this variation, making direct cross‐country cost comparisons difficult.

Cheng et al. analyzed on cost‐effectiveness of colon cancer treatment in CHs versus NCHs and demonstrated that CHs provide more cost‐effective care in the colon cancer entity.^16^ The study concluded that the financial investments associated with certification are outweighed by the improved survival of patients, meaning that treating colon cancer patients in CHs not only enhances prognosis but does so without adding a financial burden to the healthcare system. Although our study did not find such a cost‐saving outcome, we found that BC treatment in CHs had an ICER ranging from €9036 to €20,987 per LYG. This difference might stem from variations in cost structures, clinical procedures across different disease entities, and differences in costing methodologies. But both studies have consensus on the fact that cancer treatment in CHs results in substantial LYG with modest cost or cost savings.

Notably, service providers incurred a considerable cost in obtaining and maintaining DKG certification. Certification costs accounted for 13% (1.5 million euros) of the total treatment cost (11.9 million euros) per 1000 patients in the CHs group. Maintaining certification required continuous investment in quality improvement, staff education, and resource allocation. While these costs represented a financial investment for healthcare institutions, they could lead to improved patient care and clinical outcomes, as increased life years gained, potentially offsetting the initial expenditure.^15^ Larger hospitals were more likely to have advanced imaging technology readily available and to offer complex cancer treatments more frequently.^15^ Given the situation that larger hospitals were more prevalent in the certified hospital group, we adjusted our RMST estimates for age, metastasis status, comorbidities, and hospital size to reduce potential confounding from these structural differences. This adjustment slightly reduced the estimated LYG associated with treatment in CH, but the significant clinical benefit remained.

Treatment in CHs, which adhered to established quality standards and guidelines while employing a multidisciplinary approach, was associated with significant clinical benefits—measured in this study as LYG per 1000 patients. This outcome could be affected by variations in tumor characteristics between patient groups. For instance, tumor type, stage, hormone receptor status, proliferation rate, and HER2 status could differ between patients treated in CHs and NCHs. However, our insurance claims data lacked the detailed clinical information necessary to adjust for these potential differences. Using the same patient sample as our study, Schöffer et al. (from the WiZen study) linked data with the cancer registry and found no significant differences in clinical characteristics between patients treated in certified and NCHs.^12^ Using a Cox regression model with shared frailty to adjust for potential confounders, Schöffer et al. reported hazard ratios and concluded that initial treatment in CHs was associated with a statistically significant lower hazard ratio. This supported existing evidence of improved OS for BC patients in CHs. These findings are consistent with the results of our research.

Regarding BC care and treatment costs, several factors influence cost parameters. Early diagnosis and treatment can mitigate the overall economic burden by reducing the need for intensive care in later stages; approximately 90% of women diagnosed early survive at least 5 years, compared to just 15% for those diagnosed late stage.^2^ Advanced‐stage BC incurs significantly higher costs due to increased medical interventions, including palliative and end‐of‐life care.^25^ Consequently, BC‐related costs typically follow a U‐shaped pattern, peaking at diagnosis and near death, with lower costs in between.^26^ A study by Gruber et al. also observed an age‐dependent trend, finding that attributable BC costs declined with age, possibly because older patients with more comorbidities are often diagnosed at a higher stage and may shift focus to less costly palliative care rather than curative treatment.^22^ This suggests younger patients often undergo more intensive treatment.

Our study employed a bottom‐up costing approach by converting reimbursement amounts from billing codes, with inpatient admission costs reflecting the DRGs assigned. With higher per‐patient cost in CHs, which indirectly suggests that CHs generally handle more expensive treatments. While we used the national average DRG rates, actual reimbursements can vary depending on a hospital's case mix index and performance‐based payments. Larger and university hospitals typically had higher overhead costs and might receive adjusted base rates to account for specialized services and teaching responsibilities. However in the long run, larger hospitals with certifications for multiple cancer types could achieve greater efficiency as other disease entities can share the fix cost incurred by the certification process.^15^ Enhanced economic efficiency is achieved by minimizing redundant examinations, organized inter‐organ resources, and centralizing purchasing (e.g., of pharmaceuticals). This is economies of scale, a well‐established economic principle.^27^ Additionally, slight regional variations in base rates might exist due to differences in costs and other regional factors. Although the National Cancer Plan recommends receiving cancer treatment in certified facilities, over 40% of cancer patients in Germany still begin their treatment in NCHs.^14^

### 4.1 Strengths and limitations

One of the key strengths of this study is that it is the first health economic analysis comparing BC treatment in certified versus NCHs in Germany. A large dataset of 143,720 insurance claims from over 1000 hospitals tracked over a 9 years' period allowed for a detailed analysis of direct cost variables, reflecting real‐world expenditures. Unlike traditional clinical trials, this real‐world data (RWD) analysis incorporates information from routine healthcare settings, enhancing the study's external validity and making the findings more generalizable to broader patient populations.

Another major strength lies in the detailed breakdown of healthcare costs, covering inpatient, outpatient, and pharmaceutical expenditures. Billing and reimbursement systems follow standardized coding schemes (e.g., ICD, OPS, DRG, EBM, ATC, and PZN), making it easier to compare data across hospitals and regions. This granular approach is not achievable through conventional top‐down costing methods, making our findings particularly robust and informative for healthcare decision‐making.

Moreover, administrative data reflects actual healthcare utilization, costs, and treatment patterns rather than controlled clinical settings, as it captures all billed services, including hospital stays, outpatient visits, procedures, and medication used. It includes BC patients within a representative insurance system; it minimizes selection bias compared to voluntary study participation.

However, several limitations must be acknowledged. First, OS was used as the primary outcome measure. Unlike relative survival, OS does not differentiate between cancer‐related and non‐cancer‐related deaths. While this reduces potential misclassification bias, it also limits insights into disease‐specific survival. It reflects the combined impact of various treatment strategies, including surgery, chemotherapy, radiotherapy, and supportive care, making it a useful but broad measure of treatment effectiveness. We also acknowledge that claims data do not allow adjustment for key clinical variables such as tumor stage, receptor status, and genomic markers that are established predictors of BC outcomes. However, Schoffer et al., using linked cancer registry data, demonstrated that the compared cohorts were clinically similar.^12^ Although residual confounding cannot be entirely ruled out, the observed comparability in cancer‐registry characteristics proven in that study reduces the likelihood that unmeasured differences in tumor biology would substantially bias our survival estimates.

Secondly, patient transfers between hospitals could not be fully accounted for. Some patients initially treated in NCHs may have later received intensive care in CHs, but our analysis categorized patients based on their index treatment. This limitation may introduce slight bias in the estimated effectiveness of certification.

Third, our analysis excludes indirect costs (e.g., productivity losses, informal caregiving), meaning it does not capture the full economic burden of BC. Including these societal costs would likely change the relative cost differences associated with certification. Additionally, while NCHs may incur costs for services similar to certification requirements in CHs, these were not included due to a lack of literature. Factoring these potential costs into the non‐certified arm would likely decrease the ICER and improve the cost‐effectiveness profile for CHs.

Finally, the observational nature of this study and the complexity of hospital certification limit causal inference. Quantifying the costs associated with the multifaceted institutional interventions of DKG certification is challenging. The Prognos AG report is the only cost reference available on this topic. Our reliance on administrative data from SHI in a non‐randomized cohort design prevents definitive causal conclusions. While our sample from AOK SHI may not be fully generalizable to the entire German population (e.g., those with private insurance or in different regions), its relatively large size suggests our estimates are likely representative at the population level.

## 5 CONCLUSION

Our cost‐effectiveness analysis reveals that CHs provide substantial clinical benefits for BC patients with a modest incremental cost. With an ICER of €9036 per LYG, the marginal increase in healthcare expenditure associated with certification is justified by the significant survival improvements observed compared to NCHs. Our study provides compelling evidence for the economic value of DKG certification in BC care. These findings highlight the importance of certification in ensuring high‐quality, guideline‐adherent cancer care while also informing healthcare policymakers and insurers about the financial implications of certification. While our results support the continued investment in cancer center certification, further research is needed to explore its cost‐effectiveness across other cancer types and healthcare settings.

## AUTHOR CONTRIBUTIONS

**Min‐Wai Lwin:** Conceptualization; methodology; investigation; formal analysis; visualization; project administration; writing – original draft; writing – review and editing; validation; data curation; software. **Olaf Schoffer:** Conceptualization; methodology; data curation; supervision; funding acquisition; project administration; writing – review and editing; validation; investigation; formal analysis. **Christoph Streissnig:** Software; data curation; writing – review and editing; methodology. **Pauline Wimberger:** Writing – review and editing; data curation. **Michael Gerken:** Data curation; writing – review and editing. **Veronika Bierbaum:** Writing – review and editing; data curation. **Christoph Bobeth:** Data curation; writing – review and editing. **Martin Rößler:** Writing – review and editing; data curation. **Patrik Dröge:** Writing – review and editing; data curation. **Thomas Ruhnke:** Data curation; writing – review and editing. **Christian Günster:** Writing – review and editing; data curation. **Kees Kleihues‐van Tol:** Writing – review and editing; data curation. **Theresa Link:** Data curation; writing – review and editing. **Anton Scharl:** Writing – review and editing; data curation. **Elisabeth C. Sturm‐Inwald:** Writing – review and editing; data curation. **Karin Kast:** Writing – review and editing; data curation. **Thomas Papathemelis:** Writing – review and editing; data curation. **Olaf Ortmann:** Writing – review and editing; data curation. **Monika Klinkhammer‐Schalke:** Data curation; writing – review and editing. **Jochen Schmitt:** Conceptualization; methodology; data curation; supervision; funding acquisition; project administration; writing – review and editing; investigation; validation; formal analysis. **Michael Schlander:** Conceptualization; methodology; data curation; supervision; project administration; writing – review and editing; funding acquisition; investigation; validation; formal analysis.

## FUNDING INFORMATION

The main study, WiZen, was funded by the Innovation Fund of the German Federal Joint Committee (Gemeinsamer Bundesausschuss, G‐BA) (Funding number: 01 VSF17020). It analyzed both administrative and cancer registry data across 11 disease areas. This funding ensured the authors' independence in designing, interpreting, writing, and publishing the report. First and corresponding authors were employed by the public funding from the German Cancer Research Center.

## CONFLICT OF INTEREST STATEMENT

The authors declare that the research was conducted in the absence of any commercial or financial relationships that could be construed as a potential conflict of interest. Independently of this study, Jochen Schmitt received institutional grants for investigator‐initiated research from the G‐BA, BMG, BMBF, the EU, the German Federal State of Saxony, Novartis, Sanofi, ALK, and Pfizer. He also served as a paid consultant on advisory boards for Sanofi, Lilly, and ALK. Jochen Schmitt is a member of the Expert Council on Health and Care at the Federal Ministry of Health. Olaf Schoffer is employed at a university hospital with certified cancer centers and received grants from the Innovation Fund of the Federal Joint Committee for the WiZen study. He is a member of the certification committee “Skin Cancer Centers” of the German Cancer Society and a member of the expert panel for the project “Research into criteria to evaluate certificates and quality seals in accordance with Sec. 137a para. 3 sentence 2 No. 7 SGB V” at the Institute for Quality Assurance and Transparency in Healthcare (IQTIG). Independently of this study, Olaf Schoffer was a paid consultant for Novartis. Pauline Wimberger leads the DKG‐certified Breast and Gynaecological Cancer Center at the university Hospital of Dresden University of Technology and is an additional member of the Board of Directors of NCT Dresden. Pauline Wimberger receives institutional grants for investigator‐initiated research from the DFG, Krebshilfe, Sächsische Aufbaubank (SAB), Gynäko‐Onkologische Forschungsstiftung, Amgen, AstraZeneca, MSD, Novartis, Pfizer, Roche, Clovis, Abbvie, Daiichi Sankyo, Gilead and GSK. Pauline Wimberger receives honoraria as an advisory board member for Amgen, AstraZeneca, MSD, Novartis, Pfizer, Lilly, Roche, Abbvie, Eisai, Gilead, GSK, and Daiichi Sankyo. Olaf Ortmann is a member of the Executive Board of the German Cancer Society, the head of the University Cancer Center Regensburg, and a member of the Board of Directors of CCC WERA. Theresa Link works in a certified breast and gynecological cancer center. Theresa Link has received honoraria (lectures, consultancy work, and travel costs) from Novartis, Roche, Amgen, GSK, Pfizer, Gilead, Daiichi Sankyo, AstraZeneca, Lilly, Myriad, MSD, and Esai. The remaining authors declare no conflict of interest.

## ETHICS STATEMENT

The WiZen study received ethical approval from the Ethics Commission of Dresden University of Technology (reference number: EK95022019) and was registered with ClinicalTrials.gov (ID: NCT04334239). Data processing and analysis complied with the Declaration of Helsinki and the EU's General Data Protection Regulation (GDPR).

## Supporting information


**Data S1.** Supporting Information.

## Data Availability

All processed data presented in the study are included in the article and Supporting Information S1. Further information and enquiries can be directed to the corresponding author.
