# Supplementary material for: A cost‐effectiveness analysis of breast cancer treatment in certified versus non‐certified hospitals in Germany
Source: Int J Cancer. 2026 Mar 9;159(1):159–72. doi: 10.1002/ijc.70388 (PMC13140064; doi:10.1002/ijc.70388)
Supplement: Supplementary file 1 — Data S1. Supporting Information. [file IJC-159-159-s001.pdf]

Supplementary Materials: A Cost-Effectiveness Analysis of Breast Cancer Treatment in Certified vs Non-certified Hospitals in Germany

Min-Wai Lwin, Olaf Schoffer, Christoph Streissnig, Pauline Wimberger, Michael Gerken, Veronika Bierbaum, Christoph Bobeth, Martin Rößler, Patrik Dröge, Thomas Ruhnke, Christian Günster, Kees Kleihues-van Tol, Theresa Link, Anton Scharl, Elisabeth C. Sturm-Inwald, Karin Kast, Thomas Papathemelis, Olaf Ortmann, Monika Klinkhammer-Schalke, Jochen Schmitt, Michael Schlander

Table of Contents

Appendix A. Data Preparation for Obtaining the Sample for Analysis

Appendix B. DRGs for Breast Cancer: Descriptions and Counts by Certification Status

Appendix C. EBM Codes for Calculating Outpatient Service Costs

Appendix D. Issued Breast Cancer Medications and Associated Costs by Certification Status

Appendix E. Certification-Related Additional Services and Costs (Hölterhoff et al.) - Fixed and Variable Costs by Center Type.

Appendix F. Probabilistic Sensitivity Analysis

Appendix G. Probability of Cost Effectiveness

Appendix H. Summary of PSA Results: Incremental Costs, Life-Years Gained, ICER, and Cost-Effectiveness Probabilities at WTP Thresholds

Appendix A. Data Preparation for Obtaining the Sample for Analysis

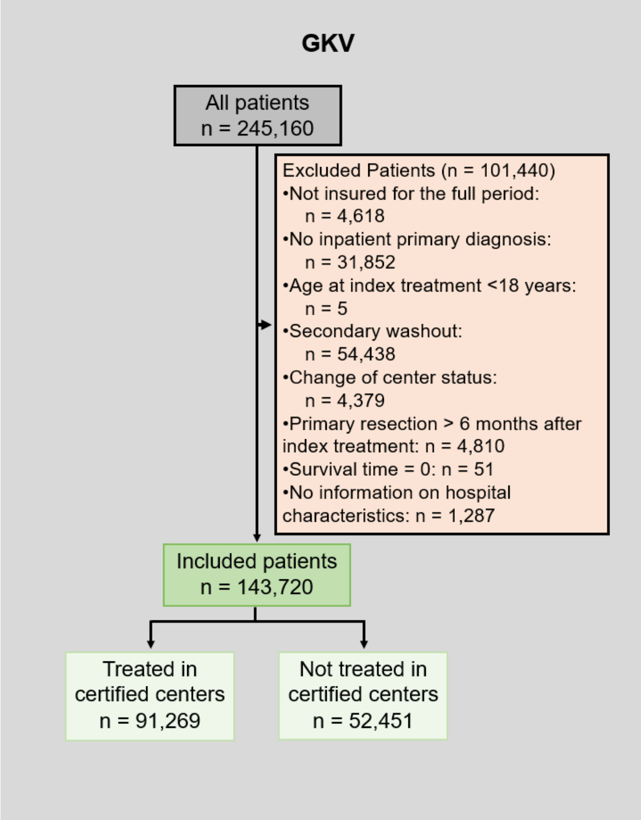

Supplementary Figure 1. Flowchart of Data Preparation for Obtaining the Sample for Analysis.

Note: The figure was previously published in Schoffer O, Wimberger P, Gerken M, Bierbaum V, Bobeth C, Rößler M, et al. Treatment in certified breast cancer centers improves chances of survival of patients with breast cancer: evidence based on health care data from the WiZen Study. Geburtshilfe und Frauenheilkunde. 2024;84(2):153–163.

Appendix B. DRGs for Breast Cancer: Descriptions and Counts by Certification Status

DRG	DRG - flat rate description	Center certification status		Total
		NCC	CC	
J23Z	Major operations on the breast in the case of a malignant neoplasm without complex surgery, without specific intervention on the female genital organs in the case of a malignant neoplasm	22939	40469	63408
J07B	Minor operations on the breast with axillary lymph node excision or extremely severe or severe CC in malignant neoplasms, without bilateral surgery, without surgery on the ovary	15218	32880	48098
J62B	Mammary malignant neoplasms, one day of occupancy or without extremely severe CC	16462	19340	35802
J25Z	Minor breast procedures for malignant neoplasms without extremely severe or severe CC	9504	20244	29748
J11B	Other procedures on the skin, subcutaneous tissue and breast without specific intervention in case of complicated diagnosis, except for para-/tetraplegia, without selective embolization in case of hemangioma, with moderately complex procedure or complex diagnosis	2964	4759	7723
J62A	Malignant neoplasms of the breast, more than one day of occupancy, with extremely severe CC	3161	3787	6948
J06Z	Mastectomy with prosthesis implantation and plastic surgery for malignant neoplasm	1635	4126	5761
J16Z	Bilateral mastectomy for malignant neoplasm or radiotherapy with surgical procedure for diseases and disorders of the skin, Subcutaneous tissue and breast, without bilateral prosthesis implantation	1810	3246	5056
J07A	Minor breast surgery with axillary lymph node excision or extremely severe or severe CC in malignant neoplasm, with bilateral surgery	1194	2321	3515
J65Z	Injury to the skin, Subcutaneous tissue and breast	978	1441	2419
J11C	Other procedures on the skin, subcutaneous tissue and breast without complicating diagnosis, except for paraplegia/tetraplegia, without selective embolization for hemangioma, without moderately complex procedure, without complex diagnosis, with specific intervention	1058	1358	2416
J14B	Plastic reconstruction of the breast in the case of a malignant neoplasm without complex reconstruction	792	1210	2002
J24D	Interventions on the breast, except for malignant neoplasms, without extensive surgery, without complex surgery	811	1188	1999
J17Z	Radiation therapy for diseases and disorders of the skin, Subcutaneous tissue and breast, irradiation for at least 9 days	623	1056	1679
J18Z	Other radiation therapy for diseases and disorders of the skin, Subcutaneous tissue and breast, more than one day of use	542	896	1438
J67Z	Breast diseases other than malignant neoplasms or mild to moderate skin diseases	439	897	1336
J14A	Plastic reconstruction of the breast with BNB with complex. Reconstruction or both sides. Mastectomy with BNB or radiation therapy with surgery. Procedure in case of illness and disorders of the skin, Subcutaneous tissue and breast, on both sides prosthesis implant or Implant a skin expander	412	885	1297
J01Z	Tissue transplantation with microvascular anastomosis for malignant neoplasms on the skin, Subcutaneous tissue and breast	326	780	1106
J67B	Mild to moderate skin diseases without CC or breast diseases other than malignant neoplasm	404	525	929
J16A	Bilateral mastectomy for malignant neoplasm	300	606	906
J16B	Radiation therapy with a surgical procedure for diseases and disorders of the skin, Subcutaneous tissue and breast	275	626	901
J65A	Injury to the skin, Subcutaneous tissue and breast, age > 70 years or severe CC	415	484	899
J08B	Other skin grafting or debridement without complex procedure, with specific intervention on skin, Subcutaneous tissue and breast, with extremely severe CC	334	502	836

J44Z	Geriatric early rehabilitation complex treatment for diseases and disorders of the skin, Subcutaneous tissue and breast	233	299	532
J08C	Other skin grafting or debridement without a complex procedure, with a complex diagnosis or with head and neck surgery, without specific skin, subcutaneous and breast surgery, or without extremely severe CC	192	339	531
J24B	Interventions on the breast except for malignant neoplasms with extensive surgery, without prosthesis implantation, without certain reduction mammoplasty	169	362	531
J10A	Plastic surgery on the skin, Subcutaneous tissue and breast for malignant neoplasms	121	305	426
J24A	Interventions on the breast except for malignant neoplasms with extensive surgery, with prosthesis implantation or certain reduction mammoplasty	156	262	418
J24C	Interventions on the breast, except for malignant neoplasms, without extensive surgery, with complex surgery	135	209	344
J26Z	Plastic reconstruction of the breast with complex skin transplantation or major surgery on the breast in the case of a malignant neoplasm with complex surgery or specific surgery on the female genital organs in the case of a malignant neoplasm	80	141	221
J11A	Other interventions on the skin, subcutaneous tissue and breast with specific intervention in case of complicated diagnosis or in case of paraplegia/tetraplegia or selective embolization in case of hemangioma	57	160	217
J10B	Plastic operations on the skin, subcutaneous tissue and breast, except for malignant neoplasms	66	129	195
J65B	Injury to the skin, Subcutaneous tissue and breast, age < 71 years, without severe CC	24	39	63
J18B	Radiation therapy for diseases and disorders of the skin, Subcutaneous tissue and breast, more than one day of occupancy, radiation on less than 5 days, less than 10 radiation treatments	15	42	57
J77Z	Complex treatment for multi-resistant pathogens in diseases and disorders of the skin, Subcutaneous tissue and breast	11	42	53
J11D	Other procedures on the skin, subcutaneous tissue and breast without a complicating diagnosis, except for para-/tetraplegia, without selective embolization for hemangioma, without a moderately complex procedure, without a complex diagnosis, without a specific procedure	21	20	41
J18A	Radiation therapy for diseases and disorders of the skin, Subcutaneous tissue and breast, more than one day of occupancy, radiation on at least 5 days or at least 10 radiation treatments	12	27	39
J35Z	Complex vacuum treatment for diseases and disorders of the skin, Subcutaneous tissue and breast	12	12	24
	Total	83900	146014	229914

Note: The Institute for the Hospital Remuneration System (InEK) annually publishes the Fallpauschalen_Katalog, a fixed amount catalog that serves as the cost reference for reimbursement to hospitals. To identify breast cancer-related DRGs and corresponding inpatient admissions for this study, we employed search terms including "breast," "mastectomy," and "mammary," resulting in a selection of 38 DRGs. Each DRG correspond to reimbursement of an inpatient admission in the Cohort.

Appendix C. EBM Codes for Calculating Outpatient Service Costs

The Uniform Evaluation Standard (Einheitlicher Bewertungsmaßstab - EBM) is a standardized framework for billing outpatient medical services within Germany's Statutory Health Insurance (SHI) system. Published quarterly by the National Association of Statutory Health Insurance Physicians (Kassenärztliche Bundesvereinigung - KBV), the EBM catalog serves as a cost reference for reimbursable outpatient services. It include the Operations and Procedure Classification System (Operationen- und Prozedurenschlüssel - OPS) tables, which standardizes the descriptions of medical procedures. The OPS table for breast cancer operations lists 130 relevant OPS codes, their corresponding EBM codes, and the allowable outpatient health services. We also employed a search terms including "breast," "mastectomy," and "mammary," to identify other non-operational EBM codes related to breast cancer.

A total of 69 EBM codes were identified as relevant to breast cancer-related outpatient services, based on the 2017 Q4 EBM catalog. These codes cover a range of diagnostic, therapeutic, and follow-up procedures essential for managing breast cancer patients within the outpatient setting. The identified EBM codes include **33041, 33091, 08320, 11440, 34270, 34271, 34272, 34273, 34274, 34275, 34430, 34431, 34700, 34701, 34702, 34703, 17362, 17363, 31111, 31112, 31113, 31114, 31115, 31116, 31117, 31118, 36111, 36112, 36113, 36114, 36115, 36116, 36117, 36118, 31501, 31502, 31503, 31504, 31505, 31506, 31507, 36501, 36502, 36503, 31202, 36202, 31211, 36211, 31212, 36212, 31601, 31608, 31609, 31632, 31633, 31821, 36821, 31822, 36822, 51040, 51041, 07345, 08345, 09345, 10345, 13435, 13675, 15345, and 26315**.

Please refer to https://www.kbv.de/html/ebm-archiv.php for EBM catalog, points and costs of each health service. These codes provide a standard amount to outpatient care reimbursement under the statutory health insurance system, facilitating appropriate and consistent billing for breast cancer-related medical services.

Appendix D. Issued Breast Cancer Medications and Associated Costs by Certification Status

Breast Cancer medication	Hospital certification status				Total Issued Medications	Total Cost of Issued Breast Cancer Medications
	CHs		NCHs			
	Number of Medications Issued	Total Cost of Medications	Number of Medications Issued	Total Cost of Medications		
**Endocrine therapy**						
Tamoxifen	315,814	7,050,366 €	188,665	4,205,856 €	504,479	11,256,221 €
Anastrozol	170,469	9,519,807 €	110,007	6,137,105 €	280,476	15,656,912 €
Letrozol	178,319	9,221,265 €	83,821	4,298,591 €	262,140	13,519,856 €
Exemestan	57,284	6,888,855 €	31,521	3,778,434 €	88,805	10,667,289 €
Fulvestrant	14,704	5,371,658 €	9,386	3,419,839 €	24,090	8,791,497 €
Goserelin	11,810	4,874,888 €	6,157	2,382,066 €	17,967	7,256,954 €
Leuprorelin	4,912	2,371,744 €	2,955	1,330,703 €	7,867	3,702,446 €
**Chemotherapeutics**						
Capecitabin	8,111	1,051,526 €	5,895	780,878 €	14,006	1,832,405 €
Vinorelbin	1,279	474,451 €	1,031	351,035 €	2,310	825,486 €
Cyclophosphamid	358	14,305 €	326	13,075 €	684	27,380 €
Doxorubicin	15	2,182 €	142	8,980 €	157	11,162 €
Docetaxel			140	24,158 €	140	24,158 €
Mitomycin	79	54,180 €	99	57,820 €	178	112,000 €
Methotrexat	151	25,048 €	77	13,605 €	228	38,654 €
Other Chemo	147	8,025 €	175	25,267 €	322	33,292 €
**Targeted Drugs**						
trastuzumab	9,499	22,886,900 €	5,493	11,245,341 €	14,992	34,132,241 €
Lapatinib	2,709	4,914,966 €	1,647	2,988,169 €	4,356	7,903,134 €
Palbociclib	1,574	2,966,817 €	1,004	1,892,430 €	2,578	4,859,246 €
Everolimus	1,943	8,289,872 €	988	4,276,172 €	2,931	12,566,043 €
Bevacizumab	241	284,454 €	141	182,357 €	382	466,811 €
Trastuzumab-Emtansin	20	38,790 €	88	170,675 €	108	209,465 €
Pertuzumab	106	294,623 €	39	108,399 €	145	403,022 €
Ribociclib	29	71,820 €	11	30,051 €	40	101,871 €
**Grand Total Cost**		**86,676,540 €**		**47,721,005 €**		**134,397,545 €**
**Average Cost per Patient**		**949.68 €**		**909.82 €**		**935.13 €**

Note: CHs = Certified hospitals; NCHs = Non-certified hospitals. The table provides a detailed breakdown of BC medications issued during a 5-year survival time of the patients and the associated costs for each medication category, differentiated by certification status. Each issued medication were recorded with ATC and PZN codes in the insurance claim dataset, which were translated to fixed price amounts. The reimbursed medication costs are adjusted to 2024 Euro.

Appendix E: Certification-Related Additional Services and Costs (Hölterhoff et al.) - Fixed and Variable Costs by Center Type.

Nr.[a]	Additional services	CC (n = 2059)		C (n = 150)		Assumed Direct Impact on Patient Survival
		Fixed cost	Variable cost	Fixed cost	Variable cost[b]	
M.1	Coordination of the center	400,221 €		14,578 €		
M.2	Centre manual	19,565 €		0 €		
M.3	Guideline implementation	22,430 €		3,797 €		Yes
M.4	Standard Operating Procedures (SOPs) development and maintenance	60,744 €		2,761 €		Yes
M.6	Patient pathways development and maintenance	29,776 €		7,053 €		Yes
M.8	Professional training events for network partners	112,462 €		6,965 €		
M.10	Referral survey	5,626 €		893 €		
M.11	Public relations, patient events and information	99,766 €		8,321 €		Yes
M.12	Patient survey	10,004 €		589 €		Yes
M.14	Patient support groups	17,163 €		2,597 €		Yes
M.15	Consultation with social workers		115,433 €			Yes
M.16	Psycho-oncological consultation		227,898 €			Yes
M.17	Palliative care coordination	79,331 €		12,876 €		Yes
M.18	Professional training / Continuous education	227,436 €		15,773 €		Yes
M.19	Continuous education for oncological specialty staff	55,458 €		7,871 €		Yes
M.20	Tumor conference		420,853 €			Yes
M.21	Feedback system	36,007 €		3,982 €		Yes
M.22	Morbidity and mortality (M&M) conferences	42,230 €		3,649 €		Yes
M.23	Quality control circle	78,699 €		4,244 €		Yes
M.24	Tumor documentation system		246,729 €			
M.25	Study management	338,503 €		12,446 €		
M.28	Specialty consultation		281,606 €			Yes
M.29	Consultation for genetic screening		36,731 €			

M.30	Particular procedure: stoma care (Breast, Colon, head-neck)		106,958 €			Yes
M.33	(Re-)certification and (re-)assessment	104,319 €		14,855 €		
Total		**1,739,742 €**	**1,436,207 €**	**123,248 €**	**101,746 €**	
Certification attributable cost per Patient		**844.95 €**	**697.53 €**	**821.66 €**	**678.31 €**	

Note: CC: oncological centers; C: organ cancer centers, the cost estimates are adjusted to 2024 Euro.
(a) The original identifying number given to each service included in the costing analysis in the Prognos report by Hölterhoff et al.
(b) Due to small sample size, the Prognos AG report did not use standard methods to estimate variable costs per BC cancer patient in CCs. Instead, they approximated variable costs by applying the fixed cost ratio between CCs and Cs (14x).
(c) Services M5, M7, M9, M27, M31, and M32 are excluded as they are exclusive to CCC and irrelevant to this analysis.

Reference

Hölterhoff M KR, Anders M, Henkel M, Resnischek C, Riedel W, Vollmer J. Benefits, Additional Costs, and Financing of Comprehensive Cancer Centers, Cancer Centers, and Organ Cancer Centers (Nutzen, Mehraufwand und Finanzierung von Onkologischen Spitzenzentren, Onkologischen Zentren und Organkrebszentren), . Berlin/Düsseldorf: Prognos AG,; 2017.

Appendix F: Probabilistic Sensitivity Analyses (PSA)

As a Probabilistic Sensitivity Analysis (PSA), an Incremental Cost-Effectiveness Ratio (ICER) scatter plot was generated using Monte Carlo simulation. This involved repeatedly drawing 10,000 samples from predefined distributions within the 95% confidence intervals of incremental costs (gamma distribution) and life-years gained (normal distribution). The scatter plot, displayed on the Cost-Effectiveness Plane (CEP), visually represents the distribution of potential incremental costs and life years gained (LYG) from BC treatment in certified hospitals (CH) compared to non-certified Hospitals (NCH). Each point on the plot reflected an iteration of the simulation, offering insights into the variability and robustness of the estimated ICER, and aiding decision-makers in evaluating the economic and clinical impact of treatment in certified hospitals. See the Figure 4 below.

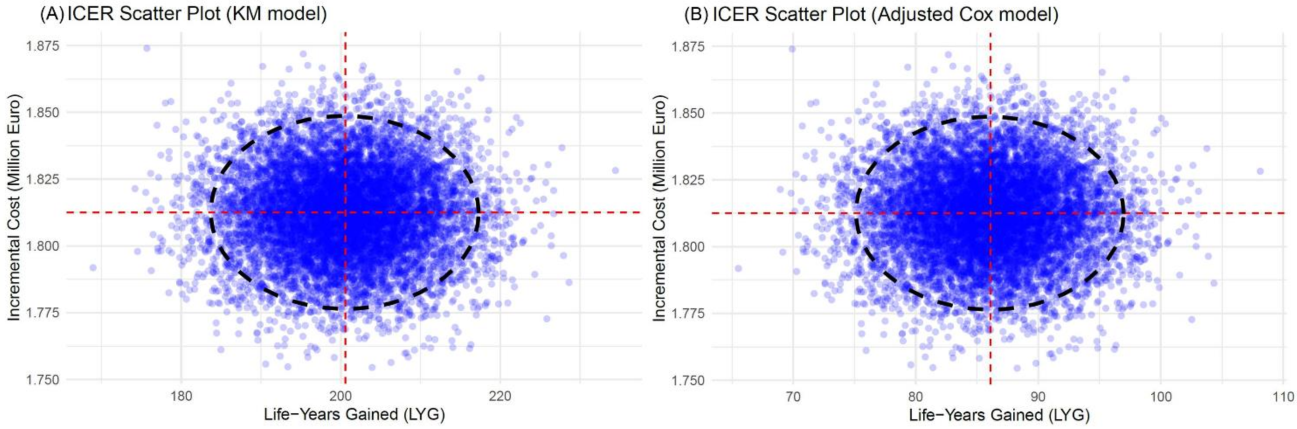

Supplementary Figure 2. ICER Scatter Plot of Breast Cancer Treatment in Certified vs Non-Certified Hospitals
(A) Kaplan–Meier Model
(B) Adjusted Cox Model

Note. This ICER scatter plot represents the probabilistic sensitivity analysis (PSA) results for BC treatment in certified (CH) versus non-certified hospitals (NCH). Each blue dot corresponds to one of 10,000 Monte Carlo simulations, depicting the incremental cost (in million euros) and life-years gained (LYG). The red dashed lines indicate the mean cost and LYG, while the black dashed ellipse represents the 95% confidence region, assuming bivariate normality. Costs have been adjusted to 2024 values using inflation indices. Results are subject to uncertainty in input parameters and model assumptions. The cost and effects are adjusted with a 3% discount rate.

Appendix G. Probability of Cost Effectiveness

To assess cost-effectiveness in relation to Willingness to Pay (WTP) thresholds during decision-making, a Cost-Effectiveness Acceptability Curve (CEAC) was generated to depict the uncertainty in cost-effectiveness analysis. The CEAC illustrates the probability that the intervention is cost-effective across a range of WTP values, providing a clearer understanding of the trade-offs between costs and benefits. This helps policymakers and stakeholders make informed decisions by quantifying the likelihood of an intervention being considered cost-effective at different thresholds.

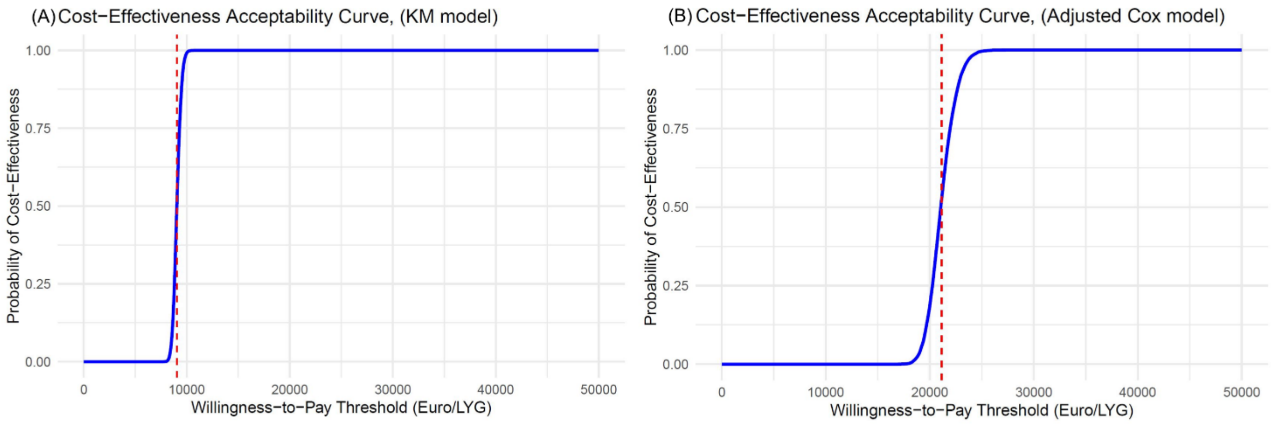

Supplementary Figure 3. Cost-Effectiveness Acceptability Curves of Breast Cancer Treatment in Certified vs Non-Certified Hospitals (Kaplan–Meier Model vs Adjusted Cox Model)
(A) Kaplan–Meier Model
(B) Adjusted Cox Model

Note: The Cost-Effectiveness Acceptability Curve (CEAC) illustrates the probability that the intervention is cost-effective at different willingness-to-pay (WTP) thresholds. The red dashed line indicates the mean ICER, where the probability of cost-effectiveness reaches 50%. As the WTP threshold increases, the probability approaches 1, suggesting higher certainty of cost-effectiveness at higher WTP levels.

Appendix H. Summary of PSA Results: Incremental Costs, Life-Years Gained, ICER, and Cost-Effectiveness Probabilities at WTP Thresholds

Outcome / Probability Level	Kaplan-Meier Model	Adjusted Cox Model
Incremental Costs (€)	1,812,481	1,812,481
Life-Years Gained (LYG)	201	86
ICER (€/LYG)	9,036	20,987
Probability of cost-effectiveness at WTP Thresholds (€ per LYG)		
25% probability	8,800	20,200
50% probability	9,000	21,100
75% probability	9,300	22,000

Notes: All cost and effect outcomes are expressed per 1,000 patients. ICER = incremental cost-effectiveness ratio (€ per LYG). Probabilities of cost-effectiveness were estimated using probabilistic sensitivity analysis, with WTP thresholds identified at the 25th, 50th, and 75th percentiles of the CEAC for each model (Kaplan–Meier and adjusted Cox).
